# Sex-dependent neurobiological features of prenatal immune activation via TLR7

**DOI:** 10.1038/s41380-018-0346-4

**Published:** 2019-01-04

**Authors:** Galen Missig, James O. Robbins, Emery L. Mokler, Kenneth M. McCullough, Staci D. Bilbo, Christopher J. McDougle, William A. Carlezon

**Affiliations:** 1 Basic Neuroscience Division, Department of Psychiatry, Harvard Medical School, McLean Hospital, Belmont, MA USA; 2 Lurie Center for Autism, Massachusetts General Hospital, Lexington, MA USA; 3 Department of Pediatrics, Harvard Medical School, Lexington, MA USA; 4 Department of Psychiatry, Harvard Medical School, Lexington, MA USA

**Keywords:** Neuroscience, Physiology

## Abstract

Immune activation during pregnancy via infection or autoimmune disease is a risk factor for neuropsychiatric illness. Mouse models of prenatal immune activation often involve maternal administration of agents that activate toll-like receptors (TLRs), a class of pattern recognition receptors that initiate innate immune responses. Such studies have focused primarily on activating the TLR3 or TLR4 subtypes, to mimic immune responses to viral or bacterial infections, respectively. Here, we characterize the effects of prenatal activation of TLR7, which is implicated in the pathogenesis of autoimmune disease. Prenatal TLR7 activation via administration of the selective agonist imiquimod (5.0 mg/kg) induces a phenotype in offspring characterized by reduced anxiety-like behavior, fragmented social behavior, and altered ultrasonic vocalization patterns at 6–12 weeks of age. The characteristics of this phenotype are readily distinguishable from—and in some ways opposite to—those seen following prenatal activation of TLR3 and/or TLR4. Prenatal TLR7-activated mice have normal baseline locomotor activity, but are hyperresponsive to stimuli including social partners, circadian cues, and gonadal hormone fluctuations. These alterations are accompanied by decreases in microglia density but increases in ramifications. RNA-sequencing of dorsal striatum, a region showing profound changes in microglial markers, indicates that prenatal TLR7 activation induces differential expression of hundreds of genes at 13 weeks of age, with virtually no overlap in differentially expressed genes between males and females. Our findings demonstrate that prenatal immune activation can promote a wide range of developmental trajectories, depending on the type and/or pattern of TLR activation and the sex of the offspring.

## Introduction

The prenatal environment profoundly influences neurodevelopment. As one example, there is accumulating evidence that inflammation during prenatal development can predispose individuals to neuropsychiatric disorders later in life [1–4]. Maternal Influenza or fever during pregnancy is associated with higher rates of autism spectrum disorder (ASD), schizophrenia (SZ), and bipolar disorder (BD) in offspring [5–7]. Similarly, maternal autoimmune disorders increase risk for neuropsychiatric disorders [8]. Data from studies in laboratory animals demonstrate that immune dysregulation can cause pathophysiology that may contribute to these elevations in risk. This is exemplified by maternal immune activation (MIA) models, in which substances that mimic infections are administered during pregnancy to examine the consequences of prenatal inflammation on neurodevelopment. Prototypical MIA models involve administration of agents that activate toll-like receptors (TLRs), which are pattern recognition receptors that initiate innate immune responses. For example, administration of either polyinosinic:polycytidylic acid (poly I:C, a TLR3 agonist) or lipopolysaccharide (LPS, a TLR4 agonist) during mid-gestation induces innate immune responses that can trigger persistent neurobiological effects in offspring [9, 10]. Maternal inflammatory responses to poly I:C or LPS can induce, through interleukin (IL)-6- and IL-17-dependent pathways, a behavioral phenotype in offspring with key features of ASD and SZ (including decreased social behavior, increased anxiety-like behavior, increased repetitive behaviors, and aberrant sensory-motor gating) in species ranging from rodents to non-human primates [11–13]. An important caveat of these types of studies is that poly I:C and LPS mimic selective aspects of a viral or bacterial response (i.e., TLR3 or TLR4 activation), whereas actual pathogen recognition and response processes occur via a diverse set of receptors and signaling mechanisms in the immune system [14], many of which have not been thoroughly studied in this context.

TLR7-mediated recognition of single-stranded RNA in endosomal compartments is integral for initiating innate immune responses to many viruses [15]. Aberrant TLR7 activation may play an important role in the pathogenesis of autoimmunity [16]. In mice, repeated administration of a TLR7 agonist produces systemic autoimmunity, characterized by the presence of anti-nuclear antibodies (ANAs) [17]. In contrast, deletion of TLR7 signaling attenuates autoimmune disease progression [18]. Mutations within the TLR7 promoter increase autoimmune disease risk, especially in males [19]. Despite its important role in immune system regulation, the ways in which aberrant TLR7 signaling impacts neurodevelopment is not understood. Here, we used a novel prenatal TLR7 activation regimen, involving administration of a selective TLR7 agonist (imiquimod), to investigate the long-term neurobiological consequences of elevated maternal TLR7 signaling during gestation.

## Methods

For more detail, see Supplemental Material 1: Extended Methods.

### Mice

C57BL/6J male and female mice aged 6–8 weeks from Jackson Laboratory (Bar Harbor, ME, USA) were time-mated. Upon successful mating, the following day was considered embryonic day 0.5 (E0.5). On E12.5, E14.5, and E16.5, pregnant dams were administered imiquimod ((IMQ), 5.0 mg/kg, subcutaneous (SC) [20]) in 10% dimethyl sulfoxide (DMSO) or vehicle (10% DMSO). Food and water were provided ad libitum. Procedures were approved by the McLean Hospital Institutional Animal Care and Use Committee in accordance with National Institutes of Health guidelines.

### Behavioral tests

**Fig. 1:**
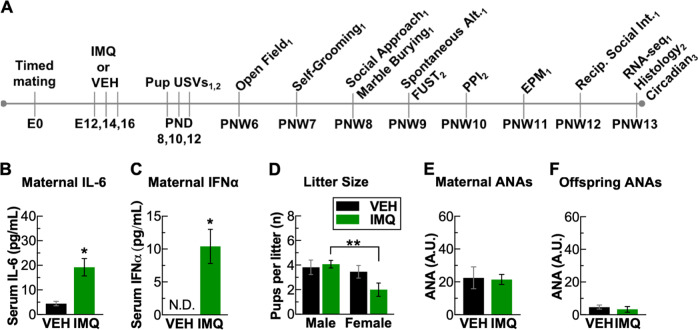
Prenatal TLR7 activation induces a maternal inflammatory response and induces a male bias in litters. **a** Experiment design showing different behavioral and physiological tests performed and the age as embryonic day (E), post-natal day (PND), or post-natal week (PNW). Experiment was run in three major cohorts denoted as subscript 1, 2, or 3. Induction of (**b**) IL-6 and (**c**) IFNα in maternal serum 3 h following last injection of IMQ on E16. **d** Prenatal IMQ led to a significant male bias in sex ratio of litters. Prenatal IMQ did not induce ANAs in (**e**) mothers or (**f**) offspring. *P < 0.05, **P < 0.01, mean ± SEM

We performed a battery of behavioral tests (Fig. 1a), comprising pup ultrasonic vocalizations (USVs), open field, self-grooming, social approach, marble burying, spontaneous alternations, female urine sniffing test, PPI, elevated plus maze, and reciprocal social interactions, similar to published reports [21, 22]. Behavior was scored by automated analysis or by an experimenter blinded to the condition of the mouse. Detailed procedures are provided in Extended Methods.

### Circadian analysis

Continuous circadian activity and temperature measurements were acquired via SC wireless telemetry transmitters (TA-F10, Data Sciences International (DSI), St. Paul, MN, USA). Transmitters were implanted according to manufacturer instructions at 11 weeks of age. Beginning at 13 weeks of age, circadian rhythms were analyzed using constant dark or “free-running” conditions [23]. In an isolated room, mice were singly housed in standard mouse cages placed upon telemetry receiver platforms (RPC-1, DSI) and entrained in 12-h light:12-h dark conditions (LD) for 7 days prior to recording a 10-day baseline using Ponemah Software (DSI). Following baseline, the lights were switched off for a 17-day recording period in constant darkness (DD). The length of the circadian period (Tau) and activity counts were calculated using Clocklab Analysis Software (Actimetrics).

### Microglia histology

Immunohistochemistry for IBA-1 using 1:1000 rabbit anti-IBA-1 antibody (019-19471, Wako Chemicals, Osaka, Japan) was performed [22]. For quantification of microglia density, whole-section montages were created using a confocal laser scanning microscope (TCS SP8, Leica, Wetzler, Germany) with Leica Application Suite software and a ×10 objective. All images were acquired under identical parameters and exported to NIH ImageJ for semi-automated analyses [24]. The identical portion of the dorsal striatum was imaged for each brain using a ×40 objective and 7-µm *z*-stacks. Maximum projections were analyzed using the Sholl Analysis tool in FIJI [25].

### RNA-seq

RNA was extracted from brain tissue punches of dorsal striatum using a column-based method [26]. Library construction, RNA-sequencing, and bioinformatic analysis was performed by Novogene (Chula Vista, CA, USA). The sequencing data from this study can be found at NCBI Sequence Read Archive (SRA) under accession PRJNA505175. Detailed procedures are provided in Extended Methods.

### Statistical analyses

Statistical analyses were performed using GraphPad Prism (version 7) or SPSS (IBM, version 24). For two groups, Student’s *t*-tests were used; for four groups, 2 × 2 (treatment × sex) ANOVAs were followed by Sidak’s-corrected multiple comparisons (VEH vs. IMQ for each sex) as pre-planned contrasts. Within-subjects data were analyzed using 2 × 2 (treatment × time) ANOVAs with repeated measures, followed by Sidak’s-corrected multiple comparisons (VEH vs. IMQ at each time point). To account for differences in variance across time, weight data was compared using a mixed model with an autoregressive covariate structure. Data was normally distributed, except in the case of USVs for which a logarithmic transformation was used for normalization. Sample sizes were chosen based on prior experiments [22] with consideration to litter effects and established guidelines [10]. A set number of offspring were randomly chosen from a litter depending on the experiment. The Grubb’s test was used for elimination of outliers.

## Results

### Prenatal TLR7 activation induces maternal inflammatory responses and fewer female offspring

The dose, 48-h injection interval, and timing of our IMQ regimen were based on an IMQ-induced model of autoimmune disease and published protocols for MIA [9, 17]. Offspring were assessed in behavioral tests of key neuropsychiatric domains before microglia histology and gene expression analyses at post-natal week 13 (Fig. 1a) (Suppl. Fig. 1A for sample sizes). IMQ administration to a separate group of pregnant dams induced an immune response, reflected by an increase of serum IL-6 3 h after the final (E16.5) IMQ injection (*t*_5_ = 3.47, *P* = 0.018) (Fig. 1b). Because TLR7 regulates the release of interferon-α (IFNα) from plasmacytoid dendritic cells [27], we also measured serum IFNα levels. IMQ treatment significantly elevated IFNα, with levels in VEH-treated dams below detection threshold (one-sample *t*-test; *t*_4_ = 4.01, *P* = 0.016) (Fig. 1c). As determined on post-natal day 8, IMQ treatment decreased litter size (*F*_1,44_ = 6.13, *P* = 0.018) and produced a sex bias in the litters, yielding half as many females (*n* = 2.0 ± 0.5 per litter) as males (*n* = 4.1 ± 0.3 per litter) (IMQ_male_ vs. IMQ_female_: *P* = 0.007) (Fig. 1d). Untoward pup loss was not observed, suggesting loss of females occurred in utero. The relative percentage of female pups (33%) is similar to a mouse model of systemic lupus erythematosus (SLE) (31% females, compared to 33%), where in utero female embryo loss was attributed to formation of cross-reactive autoantibodies to DNA [28]. As such, we measured levels of anti-nuclear autoantibodies (ANAs), which include anti-DNA autoantibodies. There were no statistically significant differences in ANA levels of IMQ-treated mothers (Fig. 1e) or offspring (Fig. 1f) compared with vehicle-exposed mothers or offspring, indicating that the female loss is unlikely directly related to formation of ANAs. Qualitatively, we did not observe overt physical malformations or signs of poor health in the IMQ-exposed pups such as excessive barbering, lethargy, or abnormal growths, but there were small (<2 g) but consistent decreases in body weight in both sexes that emerged in adulthood (treatment: *F*_1,48.07_ = 32.04, *P* < 0.01) (Suppl. Fig. 1B).

### Prenatal TLR7 activation induces multiple behavioral abnormalities

**Fig. 2:**
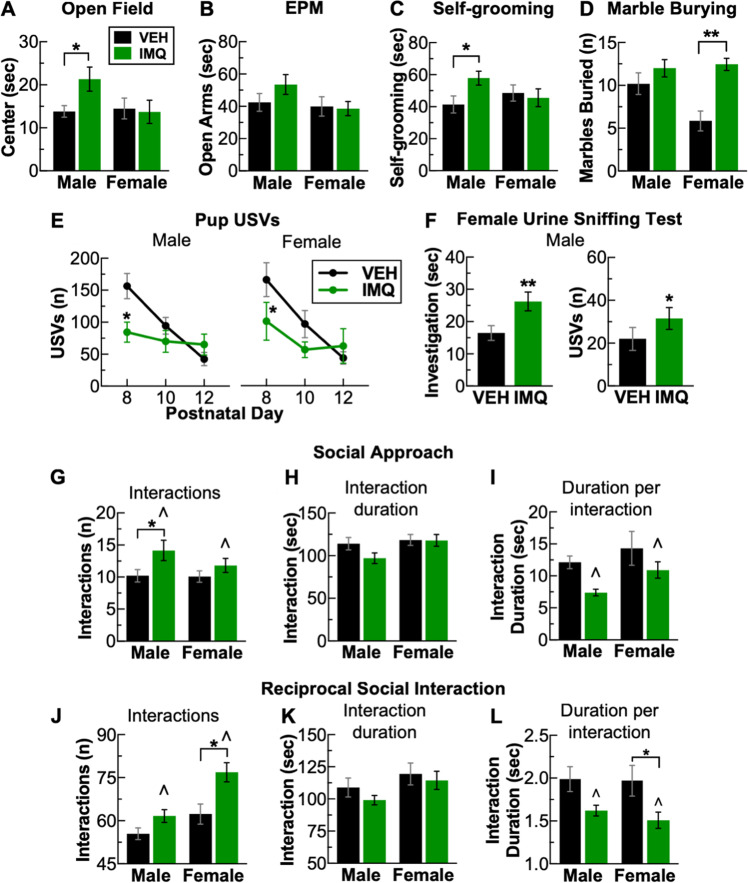
Prenatal TLR7 activation induces multiple neuropsychiatric behavioral abnormalities in a sex-specific manner. **a** Decreased center duration in the open field in male but not female mice. **b** No significant effects on open-arm time on the elevated plus maze. **c** Increased self-grooming in males but not females, however (**d**) increased marble burying in females but not males. **e** Decreased pup ultrasonic vocalizations (USVs) on post-natal day 8 for males and females. **f** Increased scent investigation (left) and USVs emitted (right) for males in the female urine sniffing test. On two different tests of social behavior, social approach (**g**–**i**) and reciprocal social interactions (**j**–**l**), mice display a “fragmented” style of social interaction characterized by **g** and **j**, an increase in the number of social interactions, (**h**) and (**k**) no change in total time interacting, (**i**) and (**l**) leading to a decrease in the time per interaction. *P < 0.05, **P < 0.01, ^∧^*P* < 0.05 (main effect of VEH vs. IMQ), mean ± SEM

**Fig. 3:**
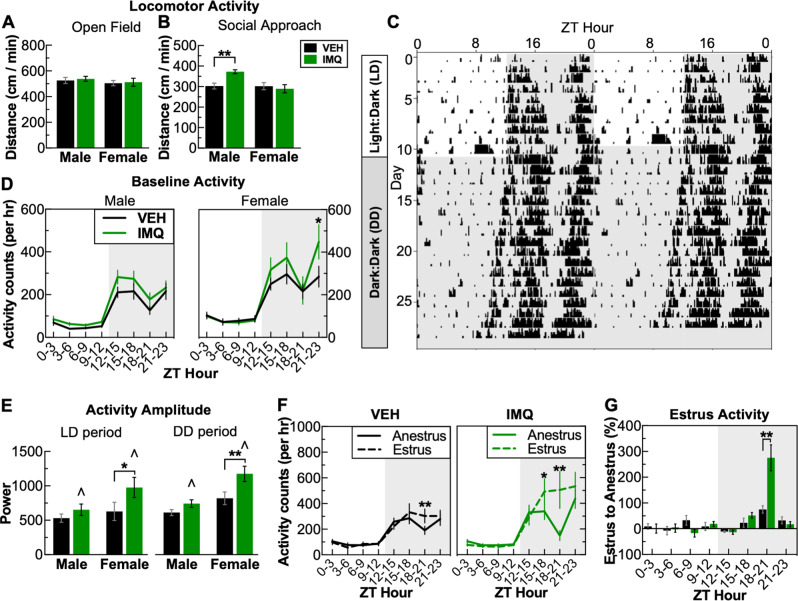
Prenatal TLR7 activation results in a reactive hyperactivity. **a** There were no changes in locomotor activity in the open field, (**b**) however, during the social approach test in the presence of a social partner, male IMQ-treated mice display locomotor hyperactivity. **c**–**g** To examine locomotor activity in detail, wireless telemetry devices were implanted to continuously record activity and temperature. **c** Double-actogram from a vehicle-treated female mouse with a 10-day baseline (12:12 h light:dark (LD), lights on zeitgeber time (ZT): 0:00, lights off: ZT 12:00, followed by 18 days of constant darkness). Note LD day 10 was excluded from analysis due to cage-change-induced activity. **d** Slight baseline elevation of locomotor activity in males and females following prenatal TLR7 activation. **e** Prenatal TLR7 activation enhanced circadian amplitude of locomotor activity. **f** Activity from VEH- and IMQ-treated females separated into anestrus and estrus phases demonstrating that estrus is characterized by increased activity during ZT 18–21. **g** Prenatal TLR7 activation led to a substantial increase in change of locomotor activity during estrus examining percent change in the activity of each mouse. *P < 0.05, **P < 0.01, ^∧^*P* < 0.05 (main effect of VEH vs. IMQ), mean ± SEM

At post-natal week 6, center times in the open-field test were higher in IMQ-exposed male (VEH_male_ vs. IMQ_male_: *P* = 0.044), but not female mice compared to VEH-exposed animals (Fig. 2a). A similar but non-statistically significant pattern emerged in the elevated plus maze test on post-natal week 11, with nominally higher open-arm times for IMQ-exposed males (Fig. 2b). These results may reflect lower levels of anxiety-like behavior, or higher levels of risk-taking behavior, in IMQ-exposed males. On post-natal week 7, there was an increase in self-grooming in IMQ-exposed male (VEH_male_ vs. IMQ_male_: *P* = 0.039) but not female mice (Fig. 2c). Conversely, on post-natal week 8 in the marble burying test, IMQ-exposed female mice buried a significantly greater number of marbles compared to VEH-exposed controls (sex × treatment: (*F*_1,51_ = 4.66, *P* = 0.036) (VEH_male_ vs. IMQ_male_: *P* = 0.004), with no effect in males (Fig. 2d). Ultrasonic vocalizations are a putative form of communication in mice [29]. On post-natal days 8, 10, and 12, pups were briefly separated from their litter and placed into a chamber containing a USV-sensitive microphone. On post-natal day 8, there were fewer USVs emitted by both IMQ-exposed male (treatment × day: F_2,74_ = 6.36, *P* = 0.003) (VEH_male_ vs. IMQ_male_: *P* = 0.039) and female pups (Treatment: F_1,29_ = 4.72, *P* = 0.038) (VEH_female_ vs. IMQ_female_: *P* = 0.035) (Fig. 2e). To examine USVs in adulthood in males, on post-natal week 9, we performed the female urine sniffing test, a putative indicator of social reward [30]. Mice were placed in an empty cage and presented with a cotton swab laced with estrus female urine, and investigation time and USVs emitted were quantified. IMQ-exposed males spent more time engaged in scent investigation (VEH_male_ vs. IMQ_male_: *t*_34_ = 2.65, *P* = 0.012) (Fig. 2f, left) and emitted more USVs (VEH_male_ vs. IMQ_male_: *t*_34_ = 2.43, *P* = 0.02) (Fig. 2f, right), suggesting increased interest and responsiveness to a rewarding social stimulus. Accordingly, we assessed social behavior in two separate tests. During post-natal week 8, we used the social approach test to examine interactions of each mouse with a conspecific control mouse enclosed in a wire mesh cage. IMQ-exposed males exhibited a distinctive pattern of interaction, characterized by higher numbers of interactions (treatment: *F*_1,48_ = 6.46, *P* = 0.019) (VEH_male_ vs. IMQ_male_: *P* = 0.032) (Fig. 2g), together with no significant change in the interaction duration (Fig. 3h), yielding shorter durations per interaction (treatment: *F*_1,48_ = 6.57, *P* = 0.013) (Fig. 2i). There were no significant effects in females. Increased numbers of brief-duration interactions may represent “fragmented” social behavior. Prior to testing with a conspecific mouse, mice were habituated in the arena in presence of an empty cage. During this period, there was a qualitatively similar (but not statistically significant) pattern of increased interactions of shorter duration investigating the empty cage, suggesting that this phenotype may generalize beyond a social stimulus (Suppl. Fig. 2A–C). During post-natal week 12, we used the reciprocal interaction test to quantify social interactions with a novel, treatment-matched conspecific allowed to freely interact. We found similar fragmentations in social behavior, except that under these conditions, IMQ exposure led to larger changes in females, with higher numbers of interactions (treatment: *F*_1,48_ = 14.51, *P* < 0.001) (VEH_female_ vs. IMQ_female_: *P* = 0.002) (Fig. 2j) and no change in interaction duration (Fig. 2h), yielding shorter interactions (treatment: *F*_1,48_ = 10.27, *P* = 0.002) (VEH_female_ vs. IMQ_female_: *P* = 0.046) (Fig. 2l). Prenatal IMQ exposure did not affect prepulse inhibition (PPI) (Suppl. Fig. 2D, E), suggesting no impairments in sensory-motor gating. Additionally, there were no effects on the spontaneous alteration test (Suppl. Fig. 2F, G), suggesting no gross alterations in short-term memory. There were no significant differences in the variance of any of these behaviors across treatment group or sex (Suppl. Fig. 2H).

### Prenatal TLR7 activation produces hyperactivity in response to stimuli

IMQ exposure led to hyperactivity under some, but not all, conditions. In the open-field test, there were no significant effects of prenatal IMQ exposure on locomotor activity (Fig. 3a). However, in the social approach test—conducted within the same arena as the open-field test—IMQ-exposed males had significantly increased locomotor activity compared to controls both with a conspecific mouse (treatment × sex, *F*_1,48_ = 7.03, *P* = 0.01) (VEH_male_ vs. IMQ_male_: *P* = 0.004) (Fig. 3b) and in presence of a novel object (empty cage) (treatment × sex, *F*_1,48_ = 7.13, *P* = 0.04) (Suppl. Fig. 3A) (VEH_male_ vs. IMQ_male_: *P* = 0.008), suggesting hyperresponsiveness was restricted to the presence of a novel stimulus. To perform a more detailed analysis of locomotor activity patterns, we examined circadian rhythm of locomotor activity in a separate cohort of mice implanted with wireless transmitters to enable continuous data collection over a period of several weeks. A 10-day baseline in 12 h light:dark (LD) conditions was followed by 17 days of constant darkness or dark:dark (DD) period to allow determinations of free-running activity and circadian rhythms without photic entrainment (Fig. 3c). Prenatal IMQ exposure did not alter the length of the circadian period (tau) in the DD period, although the effects were sex-dependent, with shorter period lengths in females compared to males (sex: *F*_1,22_  = 14.17, *P* = 0.001) (Suppl. Fig. 3B). There were no effects of IMQ exposure on weight gain (Suppl. Fig. 3C), despite reports that metabolic dysfunction is a consequence of constant darkness [31]. However, IMQ exposure led to conditional alterations in locomotor activity. For example, during the LD baseline, IMQ-exposed females had increased activity during the dark phase (treatment × time: *F*_7,70_ = 2.23, *P* = 0.042) (ZT hour 21–23 VEH_female_ vs. IMQ_female_: *P* = 0.048) (Fig. 3d). We determined the circadian amplitude of locomotor activity, which quantifies the magnitude of activity entrained to circadian rhythm. IMQ exposure led to a significant increase in amplitude (i.e., difference between peak and trough) during the baseline (LD) (treatment: *F*_1,22_ = 4.72, *P* = 0.04) that was more pronounced in females (VEH_female_ vs. IMQ_female_: *P* = 0.039), with similar effects in DD (treatment: *F*_1,22_ = 9.69, *P* = 0.005) (VEH_female_ vs. IMQ_female_: *P* = 0.005). These data indicate that IMQ-exposed mice are hyperactive in response to intrinsic circadian cues. While examining daily temperature oscillations, we noticed a prominent 4-day pattern in female mice due to the estrous cycle (Suppl. Fig. 3D). The peaks in this pattern correspond to elevations in body temperature and activity during estrus, and when combined with evidence that both of these metrics are controlled by gonadal hormones, suggest that the estrus phase can be readily determined by the presence of a monophasic temperature peak during the dark phase [32]. Utilizing the regularity of this pattern, we classified days of the LD baseline as part of either the estrus or anestrus phase. Temperature oscillations during the anestrus and estrus phase did not differ between VEH and IMQ-exposed mice (Suppl. Fig. 3E, F) and thus did not impede accurate classification. Comparing patterns between the anestrus and estrus phase indicated that the estrus phase is characterized by a substantial increase in activity levels during the dark phase for both VEH- (time × phase: *F*_7__,__35_ = 3.87, *P* = 0.003) (VEH_estrus_ vs. VEH_anestrus_: ZT hour 18–21, *P* = 0.003) and IMQ-exposed mice (time × phase: *F*_7__,__35_ = 7.32, *P* < 0.001) (IMQ_estrus_ vs. IMQ_anestrus_: ZT hour 15–18, *P* = 0.03, ZT hour 18–21, *P* < 0.0001). IMQ-exposed mice had approximately threefold higher increases in activity during the estrus phase compared to control (treatment × time: *F*_7,70_ = 8.14, *P* < 0.001) (ZT18-21: IMQ_Female_ 275.5% vs. VEH_Female_: 75.2%, *P* < 0.001) (Fig. 3g), suggesting that IMQ-exposed females are hyperactive in response to gonadal hormone fluctuations.

### Prenatal TLR7 activation reduces IBA-1 +  microglia density and alters microglia morphology

**Fig. 4:**
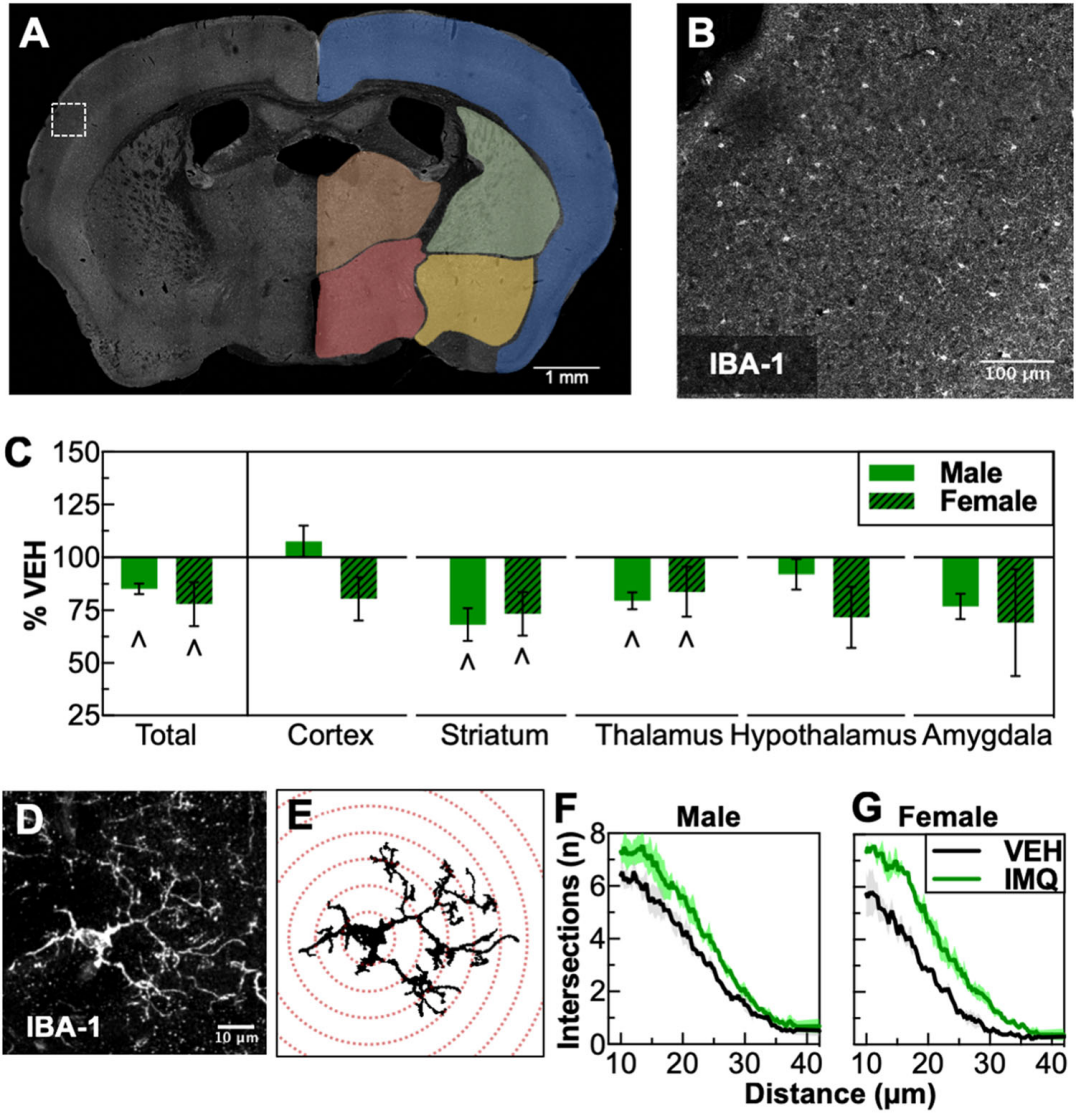
Prenatal TLR7 activation decreases microglia density and alters microglia morphology. **a** Example of a whole-section montage with different brain regions examined. **b** Inset of micrograph in **a** showing IBA-1 immunohistochemistry. **c** Decreased density of IBA-1-positive cells in several brain regions. **d** Maximum projection of a microglia cell from the dorsal striatum of a 7-µm *z*-stack from 19 images taken with a confocal microscope. **e** Graphical depiction of automated trace and Sholl analysis. **f** Results of Sholl analysis of microglia from dorsal striatum showing increased ramifications in males and (**g**) females. ^∧^*P* < 0.05 (main effect of VEH vs. IMQ), mean ± SEM

Previous reports of prenatal immune activation models using LPS (TLR4) describe long-lasting increases in microglia density, together with a less-ramified, “reactive” morphology [22, 33]. Using immunohistochemistry for IBA-1 on mid-rostral brain sections, we evaluated the effects of IMQ exposure on microglia density and morphology (Fig. 4a–c). Prenatal TLR7 activation produced decreases in the number of IBA-1+ microglia (whole brain, treatment: *F*_1,13_ = 7.72, *P* = 0.016). Decreases were most pronounced in subcortical compared to cortical regions, with significant decreases in the dorsal striatum (striatum, treatment: *F*_1,13_ = 7.78, *P* = 0.015) and thalamus (thalamus, treatment: *F*_1,13_ = 5.10, *P* = 0.042) (Fig. 4c). To determine if these reductions in number were accompanied by morphological changes, we performed a Sholl analysis to examine ramifications (Fig. 4d–g). IMQ exposure led to significant increases in branching complexity (as indicated by a rightward shift in intersections per distance) in both males (treatment × distance: *F*_141,565_ = 2.016, *P* < 0.001) (Fig. 4f) and females (treatment × distance: *F*_141,565_ = 4.53, *P* < 0.001). IMQ treatment also altered other morphological metrics, including average area per cell (treatment: *F*_1,8_ = 17.19, *P* = 0.003) (Suppl. Fig. 4A) and total number of branches (treatment: *F*_1,8_ = 8.36, *P* = 0.02) (Suppl. Fig. 4B), with no change in cell radius (Suppl. Fig. 4C). These data suggest that prenatal TLR7 activation decreases the number of IBA-1+ microglia and leads to a hyper-ramified morphology.

### Prenatal TLR7 activation induces markedly different molecular signatures in males and females

**Fig. 5:**
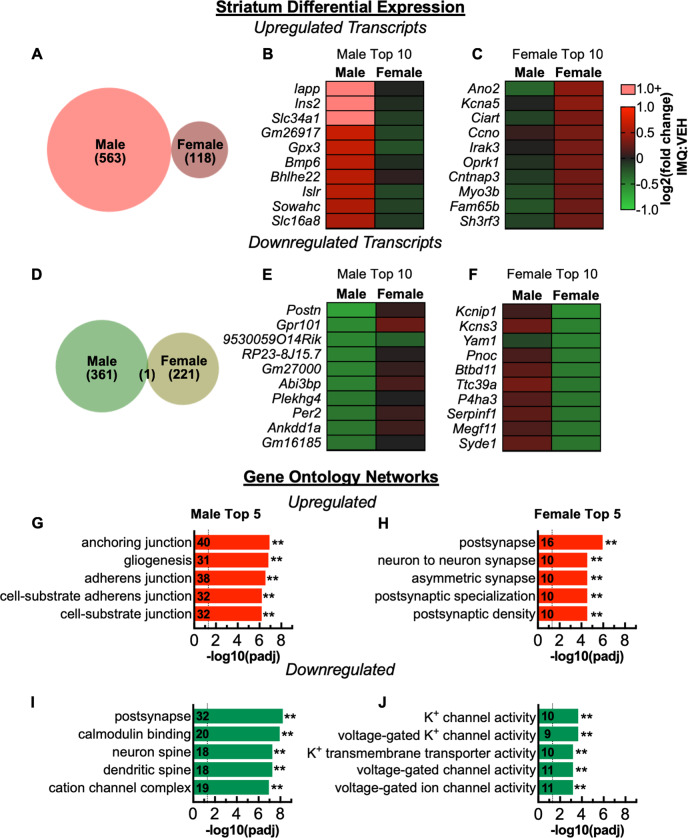
Prenatal TLR7 activation leads to a divergent molecular signature in the dorsal striatum between males and females. **a**–**f** Differentially expressed genes following prenatal TLR7 activation. **a** Proportional Venn diagram of number of significantly upregulated genes showing no overlap between males and females. **b** Top 10 (by fold change) significantly upregulated transcripts in males and (**c**) in females. **d** Proportional Venn diagram of significantly downregulated genes showing minimal overlap between males and females. **e** Top 10 significantly downregulated transcripts in males and (**f**) in females. **g**–**j** Enriched gene ontology (GO) networks. **g** GO enrichment of upregulated transcripts in males and (**h**) females. **i** GO enrichment of downregulated transcripts in males and (**j**) females. **P < 0.01

We performed RNA-seq to screen for molecular pathways affected by prenatal TLR7 activation. We focused on dorsal striatum because the alterations in microglial morphology were particularly robust and reliable in this region and established associations with neuropsychiatric disease [34, 35]. Several hundred genes were differentially expressed (*P*_adj_ < 0.05) following IMQ exposure. In males, IMQ exposure upregulated 563 and downregulated 361 mRNA transcripts. In females, fewer transcripts were affected, with 118 upregulated and 221 downregulated (Fig. 5a–f). Strikingly, there was virtually no overlap between males and females in the identity of transcripts affected by IMQ exposure (Fig. 5a, d) (Suppl. Material 2 for complete list). Furthermore, of the shared FDR significant genes affected in males and females, the direction of change was opposite for 50 of 51 (Suppl. Fig. 5A). Replication with quantitative PCR analysis was consistent with RNA-seq for the genes analyzed (Suppl. Fig. 6). We examined gene ontology (GO) enrichment to explore whether the genes affected had common or related functions. In males, IMQ exposure upregulated multiple networks related to adhesion molecules and gliogenesis (Fig. 5g), whereas it upregulated synaptic components—especially postsynaptic—in females (Fig. 5h). Transcripts downregulated by IMQ exposure were related to synaptic components in males (Fig. 5i) and ion channels in females (Fig. 5j). A GO enrichment analysis of transcripts affected in both males and females revealed that those related to synaptic components changed in opposite directions, with decreases in males but increases in females (Suppl. Fig. 5B). Comparing differentially expressed genes to two databases of cell-type-specific enrichment [36, 37] revealed that in males the highest proportion of upregulated transcripts overlap with genes enriched in oligodendrocytes, whereas in females the highest proportion is with neurons (Suppl. Figs. 7–9). Conversely, for downregulated transcripts, the highest proportion is with neurons in males and with oligodendrocytes in female (Suppl. Figs. 7–9).

## Discussion

We used a prenatal TLR7 activation regimen in mice to investigate the long-term neurobiological consequences of heighted TLR7 signaling during gestation. We discovered that prenatal immune activation selectively targeting TLR7 results in the birth of fewer female offsprings and induces numerous effects on behavior and microglia that are substantially different—and sometimes opposite to—those seen when targeting TLR3 (by poly I:C) and/or TLR4 (by LPS) [9, 22]. We determined that an underlying feature of the prenatal TLR7 activation phenotype is “conditional hyperresponsiveness,” characterized by exaggerated responses to both exteroceptive (social partner) and interoceptive (circadian cues and gonadal hormones) stimuli. Finally, we demonstrated that prenatal TLR7 activation alters expression of hundreds of genes in manners that are highly dependent on sex, with virtually no overlap between males and females. These changes occurred in response to an environmental (immune) challenge, in the absence of any genetic predispositions or vulnerabilities.

When compared to established prenatal poly I:C or LPS regimens, many of the same behavioral domains are affected by prenatal IMQ exposure, but with qualitatively different outcomes. Whereas perinatal poly I:C or LPS often increases anxiety-like behavior, we observed a decrease in this domain with prenatal IMQ [22, 38]. Further, perinatal poly I:C or LPS reportedly decreases social behavior [9, 22], which is a fundamentally different outcome than fragmentation of social behavior (increased number of interactions, but shorter in duration) following IMQ exposure. Co-occurring with these contrasting behavioral alterations are changes in microglia density and morphology that are fundamentally different from those seen after early TLR3 and/or TLR4 activation. Perinatal LPS increases IBA-1+ microglia density and results in an reactive, less-ramified morphology [22, 33], whereas we found here that IMQ exposure decreases IBA-1+ microglia density and produces more ramified morphology. These findings indicate that all forms of prenatal TLR activation are not equivalent, and that outcomes can vary substantially depending on the particular receptors and pathways activated.

One of the primary behavioral perturbations of prenatal TLR7 activation is a hyperresponsivity to various types of stimuli. This hyperresponsivity generalizes to both exteroceptive stimuli, such as a social partner or a novel object, and interoceptive stimuli, such as circadian rhythms and gonadal hormones. The mechanisms driving this hyperactivity remain unclear, with transcriptomic analysis suggesting numerous changes synaptic, glial, and metabolic alterations. However, several of these behavioral abnormalities are consistent with altered functioning of the dopaminergic system. In males, prenatal TLR7 activation induces increased scent investigation and emission of USVs to female urine, a stimulus known to induce dopamine release within the nucleus accumbens [30]. In females, prenatal TLR7 activation induces an estrus-induced hyperactivity, a feature that might also be mediated by enhanced dopaminergic function, since during estrus there is both elevated striatal dopamine signaling and enhanced behavioral responses to psychomotor stimulants [39]. An important question is whether these mice show more generalized alterations in reward-related behaviors. This question is difficult to address, considering that commonly used procedures such as the sucrose preference test are also sensitive to alterations in baseline anxiety states, which were seen in the present studies. In the future, use of procedures such as the intracranial self-stimulation (ICSS test), which is minimally sensitive to anxiety-related states [40], may provide deeper insight on this specific domain.

Some effects of prenatal TLR7 activation differ substantially between males and females, whereas some were similar. On multiple behavioral measures, males were more affected (social approach, open field, self-grooming), although effects on microglia were qualitatively similar. The most striking sex difference was in the transcriptomic profile of prenatal IMQ exposure with completely different patterns in males and females. This is broadly consistent with emerging studies suggesting dramatic differences in transcriptional responses in the brains of males and females. In mice, social defeat stress leads to altered expression of hundreds of genes in the ventral striatum, but with minimal overlap between males and females [41]. Similarly in humans, a recent meta-analysis revealed that major depressive disorder leads to vastly different transcriptional responses in the brains of men and women, and that a considerable portion of genes are altered in the opposite direction [42]. Paralleling the current findings with prenatal TLR7 activation, this opposite pattern in part was due to a decrease in synapse-related genes in men, but an increase in synapse-related genes in women [42]. Our studies suggest that males and females can mount markedly different molecular responses to immune challenge, which may contribute to sex differences in some of these conditions (e.g., ASD [43]).

There are several important caveats for the current studies. Our experiments evaluated the neurodevelopmental outcomes of heightened TLR7 signaling during gestation, based on the established role of TLR7 signaling in autoimmunity and accumulating evidence for associations between autoimmune disorders and neuropsychiatric disorders. Our studies do not model autoimmunity per se, but rather produce an autoimmune diathesis during gestation to investigate if the same conditions that lead to autoimmunity also alter neurodevelopment. Additionally, TLR7 is activated by many viruses (especially single-stranded RNA viruses, such as influenza [44] and Zika virus [45]) and as well as certain bacteria [46], such that this model also recapitulates an aspect of maternal infection. Prenatal IMQ exposure produces a selective loss of females in early development that might create a survivorship bias (i.e., the error from only being able to examine those that lived). This bias could contribute to the sex differences we observed, and one possibility is that the female embryos with the strongest response to immune challenge did not survive. Further, due to the estrus-specific alterations in locomotor activity, future studies will be needed to systematically examine the influence of the estrous cycle in behavioral and neurophysiological disruptions of prenatal IMQ exposure. Additionally, a recent study suggests that disruption of social communication between pups and mothers may adversely affect maternal care [47]. As we observed changes in pup USVs, altered in maternal care is a potential intermediate mechanism in this model. While future studies are needed to systematically identify the mechanisms of prenatal TLR7 activation, one candidate mechanism is type-I interferon signaling, considering the vast quantities released from plasmacytoid dendritic cells following TLR7 activation [27].

Considered with the existing literature, our findings suggest that early immune system activation can promote various—sometimes even opposite—neurodevelopmental trajectories, depending on the receptors and pathways activated. As such, the precise phenotype resulting from prenatal immune activation may be highly dependent on the specific immune mechanisms activated and the resulting inflammatory milieu present in the prenatal environment. The ability of prenatal immune signaling to induce diverse phenotypes might explain why maternal inflammatory conditions are broad risk factors for a variety of disorders including ASD, SZ, BD, and attention deficit-hyperactivity disorder (ADHD) [5, 6, 48, 49]. Considering the hyperresponsivity induced by prenatal TLR7 activation, this new model may enable a deeper understanding of conditions where hyperactivity is a characteristic feature (e.g., ASD, BD, ADHD). Many aspects of this phenotype parallel features of ADHD and recent evidence suggests that parental history of autoimmune disorders increases the risk of ADHD [49, 50]. Identification of neuroimmune processes that contribute to the core features of neuropsychiatric disease may lead to improved diagnosis, treatment, and prevention of these debilitating and currently intractable conditions.

## Supplementary information

Supplemental Extended Methods

Supplemental Figures

Supplemental Data
