# Supplementary material for: Sex-dependent neurobiological features of prenatal immune activation via TLR7
Source: Mol Psychiatry. 2019 Jan 4;25(10):2330–41. doi: 10.1038/s41380-018-0346-4 (PMC7515834; doi:10.1038/s41380-018-0346-4)
Supplement: Supplementary file 1 — Supplemental Extended Methods [file 41380_2018_346_MOESM1_ESM.pdf]

SUPPLEMENTAL EXTENDED METHODS

Animals

C57BL/6J male and female mice aged 6-8 weeks from Jackson Laboratory (Bar Harbor, ME, USA) were time-mated. Upon successful mating, the following day was considered embryonic day 0.5 (E0.5). On E12.5, E14.5, and E16.5, pregnant dams were administered Imiquimod ([IMQ], 5.0 mg/kg, subcutaneous [SC][20]) in 10% dimethyl sulfoxide (DMSO) or vehicle (10% DMSO). Food and water were provided *ad libitum* and cages were changed weekly, but not within 48 hours prior to each behavioral test. Timed-mated females were housed in cages with a ventilated top for the duration of pregnancy and nursing. Following weaning offspring were housed in open-air cages. Procedures were approved by the McLean Hospital Institutional Animal Care and Use Committee in accordance with National Institutes of Health guidelines.

Pup Ultrasonic Vocalizations

Maternal separation-induced pup ultrasonic vocalizations (USVs) were recorded on post-natal day 8 (PND8), PND10, and PND12. Pups were relocated from their home cage to a sound-attenuated recording chamber. Emitted USVs were recorded for 3 minutes at room temperature (21±2C) using a microphone (CM16/CMPA, Avisoft Bioacoustics, Berlin, Germany) positioned directly above the pup. The calls transmitted directly to a pre-amplifier (Avisoft Ultrasoundgate 416H, Avisoft Bioacoustics) connected to a computer that stored the sonograms. SASLab Pro software (Avisoft, Version 5.1) was used for semi-autonomous quantitative analysis of USV metrics, including call count, frequency, and duration. After 3 minutes of recording, pups were relocated to an empty cage containing bedding under a heating pad to identify which pups had been subjected to testing. The empty cage was covered with a filter-top and was on a heating pad. The acoustic chamber was cleaned with ethanol between each trial.

Open Field Test

The open field test assessed anxiety-like behavior at 6 weeks of age, in a manner similar to that previously described[30]. Mice were removed from their home cage and placed in a corner of the open field chamber (44cm length x 44cm length x 30cm height) within an isolated, brightly-lit room. A 5-minute trial was recorded with a ceiling-mounted camera and analyzed using Ethovision-XT software (Noldus, Version 7.0). Parameters included total distance traveled and the number of entries and time spent in the center of the open field chamber (14cm x 14cm). The Open Field chamber was cleaned with ethanol between each trial.

Elevated Plus Maze

The elevated plus maze (EPM) assessed anxiety-like behavior at 11 weeks of age.
Mice were removed from their home cage and placed in the center of the EPM apparatus (a cross elevated 80cm above the ground and with two open arms and two perpendicular closed arms [30cm arms with 5.5cm x 5.5cm center space] with 5.5cm walls. A 5-minute trial was recorded with a ceiling-mounted camera and analyzed using Ethovision-XT software (Noldus, Version 7.0). Parameters included distance traveled, time spent in the arms, and the number of entries into each arm. The EPM was cleaned with ethanol between each trial.

Social Approach Test

The social approach test assessed social behavior at 8 weeks of age, in a manner similar to that previously described[30]. The test consisted of two 3-minute trials in an open arena (44cm length x 44cm width x 30cm height). In the first trial the mouse was placed into the arena with an empty metal wire cage (10cm length x 8cm width x 6cm height). In the second trial, a novel age- and sex-matched conspecific mouse was placed within the metal cage. The trials were recorded with a ceiling-mounted camera and analyzed using Ethovision-XT software (Noldus, Version 7.0).

Parameters analyzed included distance traveled and number of entries and time spent in the social interaction zone, which comprised a 25cm x 12cm space centered around the social partner.

Reciprocal Social Interaction Test

The reciprocal social interaction test or dyadic social interaction test assessed social behavior at 12 weeks of age. The test consisted of placing two sex and treatment-matched mice that had not been previously exposed to each other in a large empty cage (40cm length x 20cm width x 20 cm height) and allowing them to freely interact for a 5-minute duration. To avoid territorial aggression, mice were each placed in the interaction chamber within a period of 10 seconds. These interactions were video-recorded using a ceiling mounted camera. Videos were analyzed by a blinded experimenter reviewing the video recordings at 0.5x speed using Stopwatch+ software (Center for Behavioral Neuroscience, Georgia State University) which included measuring the number of interactions and the total duration of interactions.

Marble Burying

The marble burying task assessed repetitive or stereotyped behaviors. In this task black glass marbles (1.5cm diameter) are arranged into a 5 x 4 matrix on top of bedding (Northeastern Products Corp. Alpha Chip), which fills approximately 1/2 the volume of the cage (25cm length x 16cm width x 13cm height). A subject mouse is placed in the cage, which is then covered by a filter top, and is allowed to interact freely with the marbles for a duration of 20 minutes. A marble is considered buried if bedding covers greater than 2/3 of its surface area. At 20 minutes, mice are removed from their respective cages and blinded experimenters determine the number of marbles buried.

Self-grooming

The self-grooming test assessed repetitive or stereotyped behaviors. Mice are placed individually in empty cages (25cm length x 16cm width x 13cm height) covered by a filter top. A blinded experimenter recorded the total duration of self-grooming behavior during a 10-minute trial.

Female Urine Sniffing Test

The female urine sniffing test was performed in a manner similar to that described (Malkesman et al. 2010). Briefly, a male mouse was removed from its home cage and placed in an empty clean cage and allowed to briefly habituate for 2 minutes illuminated by a dim red light. A cue tip laced in estrus female urine was suspended from the top of the cage by placing the swab in a 1 mL pipette tip from the metal grid on top of the cage. An observer blinded to treatment condition recorded the duration of investigation during the 5-minute trial. Ultrasonic vocalizations were recorded using a microphone (CM16/CMPA, Avisoft Bioacoustics, Berlin, Germany) positioned above the cage, using the same system described previously. USVs were analyzed manually with a blinded observer using Avisoft SASLab Pro software (Version 5.1) as automated scoring tended to be less accurate for the more complex calls emitted by adult mice. Urine was collected from female C57BL/6J mice on the day were identified to be in the estrus phase determined by vaginal cytology under the microscope.

Cytokine and autoantibody analysis

Analysis of cytokines and autoantibodies was performed using commercially available ELISA kits for IL-6 (R&D Systems, #M6000B), IFNα (PBL Assay Science, #42115-1), and ANAs (Alpha Diagnostic International Inc., 5210). Samples were run in duplicate according to manufacturer's instructions. Blood was collected 3 hours following the last IMQ injection or at 13 weeks of age for the offspring via cardiac puncture and then allowed to clot for 1 hour before centrifugation for serum collection.

Circadian Activity and Temperature

Circadian activity and temperature measurements were acquired using an implanted wireless telemetry device (TA-F10, Data Sciences International [DSI], St. Paul, MN, USA) that continuously recorded activity and temperature. Transmitters were implanted subcutaneously according to manufacturer's instructions at 11 weeks of age. Beginning at 13 weeks of age, circadian rhythms were analyzed using constant dark or "free-running" conditions similar to previously described protocols[31]. In an isolated room, mice were singly housed in standard mouse cages placed upon telemetry receiver platforms (RPC-1, DSI). Mice were entrained in 12-hr light:12-hr dark conditions (LD) for 7 days prior to recording a 10-day baseline using Ponemah Software (DSI). Following the baseline period the lights were switched off for a 17-day recording period in constant darkness (DD). The length of the circadian period (Tau) and activity counts were calculated using Clocklab Analysis Software (Actimetrics).

Microglia histology

Immunohistochemistry for IBA-1 using 1:1000 rabbit anti-IBA-1 antibody (019-19471, Wako Chemicals, Osaka, Japan) was performed as described[30]. For quantification of microglia density, whole section montages were created using a confocal laser scanning microscope (TCS SP8, Leica, Wetzler, Germany) with Leica Application Suite software and a 10X objective. All images were acquired under identical settings and parameters, and exported to NIH ImageJ for analysis via semi-autonomous methods. Morphological analysis was performed as described[32]. The identical portion of the dorsal striatum was imaged for each brain using a 40X objective and 8 µm z-stacks. Maximum projections were analyzed using the Sholl Analysis tool in FIJI[33].

Transcriptomic analysis

At 13 weeks of age mice were decapitated and brains were immediately extracted, rapidly frozen in dry ice cold isopentane, and stored at -80C. Bilateral tissue punches (2mm diameter) of the

dorsal striatum were collected in Eppendorf tubes on dry ice. RNA was isolated using a column-based method with PureLink RNA Mini Kit (Invitrogen, Thermo Fisher Scientific). RNA quantity and purity were analyzed using an Agilent Bioanalyzer. 1.5-3 μg of RNA were sent for RNAseq analysis and 1 μg of RNA was used for qPCR analysis. RNA samples had RIN (RNA integrity number) >8.0. Library construction, sequencing, and analysis was performed using standard analysis by Novogene. mRNA was purified from total RNA using poly-T oligo-attached magnetic beads. Library was constructed using NEBNext Ultra Library Prep Kit. Sequencing was performed on an Illumina Platform with 150 paired end reads. Each sample had 40-60M clean reads and Q30 >90%. Mapping was accomplished using STAR software with samples having >93% mapped reads. Differential expression analysis was performed using DESeq2 R package. Biological replicates had Pearson correlation coefficients >0.98. The sequencing data from this study can be found at NCBI Sequence Read Archive (SRA) under accession PRJNA505175.
