# Supplementary material for: Sex-dependent neurobiological features of prenatal immune activation via TLR7
Source: Mol Psychiatry. 2019 Jan 4;25(10):2330–41. doi: 10.1038/s41380-018-0346-4 (PMC7515834; doi:10.1038/s41380-018-0346-4)

SUPPLEMENTAL FIGURE CAPTIONS

Fig. S1: Experiment sample size and graph of body weight. A) Table of the sample sizes for each experiment including the number of litters per experiment. Behavior group 1 and 2 correspond to the groups denoted on Fig. 1A). **B)** Graph of body weight over weeks of age demonstrating a slight but significant decrease in weight gain following prenatal TLR7 activation. Weight was compared with a mixed-model with an autoregressive covariance structure. Mean ± SEM

Fig. S2: Prenatal TLR7 activation did not alter prepulse inhibition or spontaneous alternation behaviors. A) Prenatal IMQ-treatment did not change baseline startle or **B)** prepulse inhibition in either male or females. There was a significant difference between males and females in baseline startle that is potentially due to weight differences. **C)** Prenatal IMQ-treatment did not affect spontaneous alternations or **D)** total number of entries suggesting no gross impairment in short-term memory. Mean ± SEM

Fig. S3: Sex-specific alterations in circadian period and determination of estrus cycle by temperature oscillations. A) Prenatal IMQ-treatment did not change the length of the circadian period derived from the constant darkness period; however, there was a significantly shorter period overall in female mice. **B)** No significant alterations in body weight gain during the circadian experiment. **C)** Example of three traces from VEH- and IMQ-treated female mice illustrating the monophasic peak used to reliably discriminate the estrus from anestrus phase. An “E” denotes days identified as estrus phase. Only LD baseline period was used for further analysis. **D)** IMQ-treatment did not significantly alter temperature oscillations during the anestrus or **E)** estrus phase.

F) Increased activity during the dark phase during estrus in IMQ-treated mice, corresponding with changes in vehicle-treated females in Figure 3F, and shown as a change per each mouse in Figure 3G. * $P < 0.05$, ** $P < 0.01$ Mean ± SEM

Fig. S4 **Prenatal TLR7 activation affects microglia morphology.** Morphological analysis of IBA-1 positive cells in dorsal striatum derived from sholl analysis (Figure 4D-G) demonstrating prenatal TLR7 activation led to: **A)** a significant increases total area of thresholded immunoreactivity, **B)** an increase in the total number of branches derived from automated sholl analysis, **C)** no change in the average radius of the cells. * $P < 0.05$, ∧ $P < 0.05$ (main effect of VEH vs IMQ) Mean ± SEM

Fig. S5 **Opposite changes in shared differentially expressed genes between males and females following prenatal TLR7 activation.** **A)** Heatmap of significant ($P_{adj} < 0.05$) differentially expressed genes between males and females, 50 of 51 changed in the opposite direction. **B)** Gene Ontology networks of up- and downregulated shared between males and females.

Fig. S6 **RNA-seq and qPCR show comparable results.** RT-qPCR analysis from same RNA tissue samples for a selection of genes differentially expressed in RNAseq, showing correlation coefficient between qPCR fold change and RNAseq FPKM for males (*left*), females (*middle*) and the total fold change (*right*) for **A)** *Ano2*, **B)** *Gfap*, **C)** *Oprk1*, **D)** *Kcnj10*, and **E)** *Synpr*. * $P < 0.05$, ** $P < 0.01$

Fig. S7 **Cell type specific expression analysis of RNA-seq.** Proportion of differentially expressed genes from striatal transcriptomic analysis compared with top 500 genes in a cell-type specific enrichment database[37]. Results from upregulated differentially expressed transcripts from **A)** males and **B)** females and downregulated transcripts

from **C)** males and **D)** females. Total refers to number of differentially expressed genes from transcriptome analysis and number next to legend is number of overlapping transcripts that are enriched in the cell type.

Fig. S8 **Male cell type specific expression analysis of RNA-seq.** Results from a publicly accessible cell type specific expression analysis tool examining differentially expressed transcripts from male striatal transcriptomic analysis[38].

Fig. S9 **Female cell type specific expression analysis of RNA-seq.** Results from a publicly accessible cell type specific expression analysis tool examining differentially expressed transcripts from female striatal transcriptomic analysis[38].

Supplemental Figure 1

A)

Experiment	Maternal Treatment	Sex	Litters	Mice	Sections	Cells
Behavior Group 1	Vehicle	Male	5 (≤ 3 per litter)	15		
	Vehicle	Female		14		
	IMQ	Male	5 (≤ 3 per litter)	15		
	IMQ	Female		11		
Behavior Group 2	Vehicle	Male	6 (≤ 3 per litter)	18		
	Vehicle	Female		12		
	IMQ	Male	6 (≤ 3 per litter)	18		
	IMQ	Female		9		
Circadian	Vehicle	Male	4 (= 2 per litter)	8		
	Vehicle	Female		6		
	IMQ	Male	3 (= 2 per litter)	6		
	IMQ	Female		6		
IHC IBA-1	Vehicle	Male	3 (≤ 2 per litter)	5	3	
	Vehicle	Female		3	3	
	IMQ	Male	4 (≤ 2 per litter)	5	3	
	IMQ	Female		4	3	
IHC IBA-1 Morphology	Vehicle	Male	3 (= 1 per litter)	3	3	64
	Vehicle	Female		3	3	61
	IMQ	Male	3 (= 1 per litter)	3	3	60
	IMQ	Female		3	3	56
RNA-seq	Vehicle	Male	3 (= 2 per litter)	6		
	Vehicle	Female		6		
	IMQ	Male	3 (= 2 per litter)	6		
	IMQ	Female		6		

B)

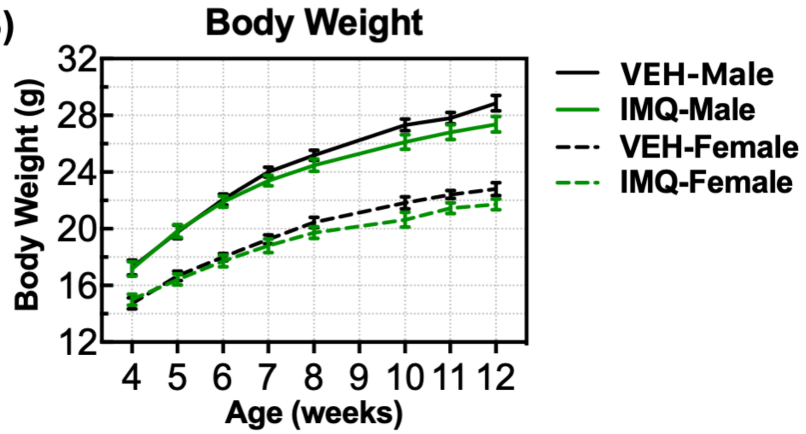

Supplemental Figure 2

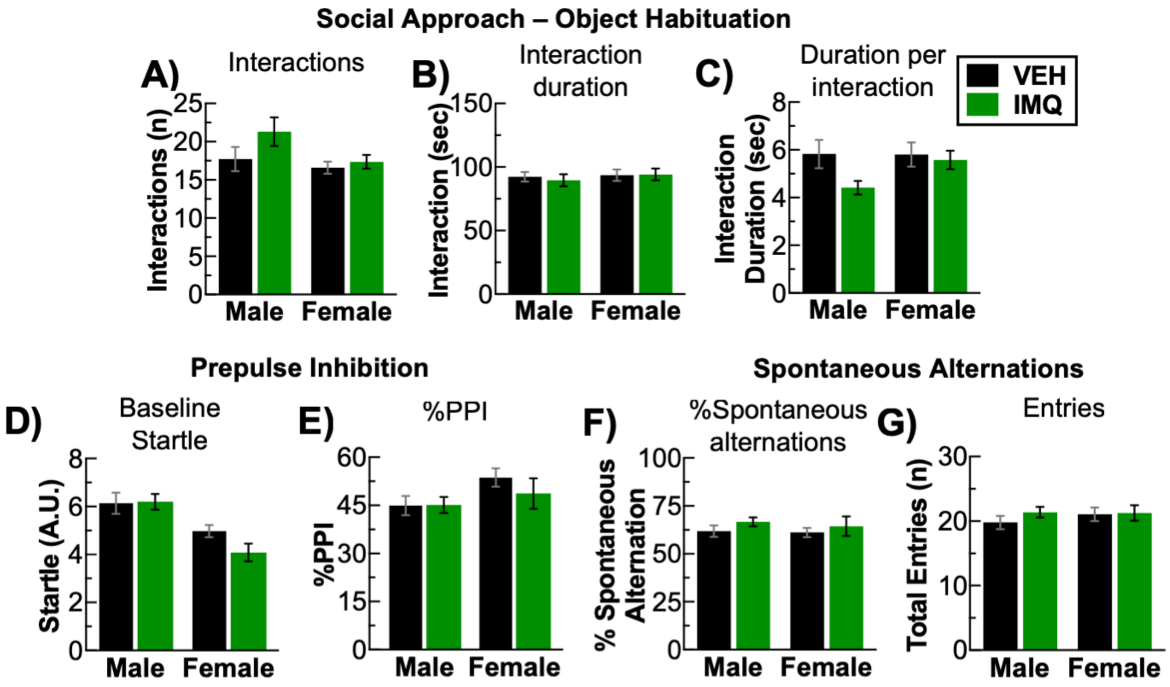

H) **Variance of Behavioral Tests (Brown-Forsythe Test)**

Open Field
Distance (cm/min): $F_{3,50}=0.30$, $P=0.83$
Center (sec): $F_{3,50}=1.31$, $P=0.28$

Self grooming
$F_{3,51}=0.14$, $P=0.94$

Social Approach
Interactions (n): $F_{3,48}=1.54$, $P=0.22$
Interactions (sec): $F_{3,48}=0.35$, $P=0.79$
Avg. Duration per Interaction (sec): $F_{3,48}=1.77$, $P=0.17$
Distance: $F_{3,48}=1.01$, $P=0.39$

Social Approach – Object Habituation
Interactions (n): $F_{3,48}=2.15$, $P=0.11$)
(Interactions (sec): $F_{3,48}=2.01$, $P=0.13$)
(Avg Duration per Interaction): $F_{3,48}=1.98$, $P=0.13$)
(Distance: $F_{3,48}=0.91$, $P=0.44$)

Marble burying
$F_{3,51}=1.24$, $P=0.31$

Spontaneous Alternations
$F_{3,49}=0.35$, $P=0.79$

Prepulse Inhibition
(Startle: $F_{3,50}=1.96$, $P=0.13$)
(PPI: $F_{3,50}=0.23$, $P=0.23$)

EPM
Open arm (sec): $F_{3,50}=0.23$, $P=0.88$

Reciprocal Social Interaction
(Interactions (n): $F_{3,48}=1.18$, $P=0.32$)
(Interactions (s): $F_{3,48}=1.28$, $P=0.29$)
(Avg Duration per Interaction): $F_{3,48}=2.02$, $P=0.12$)

Supplemental Figure 3

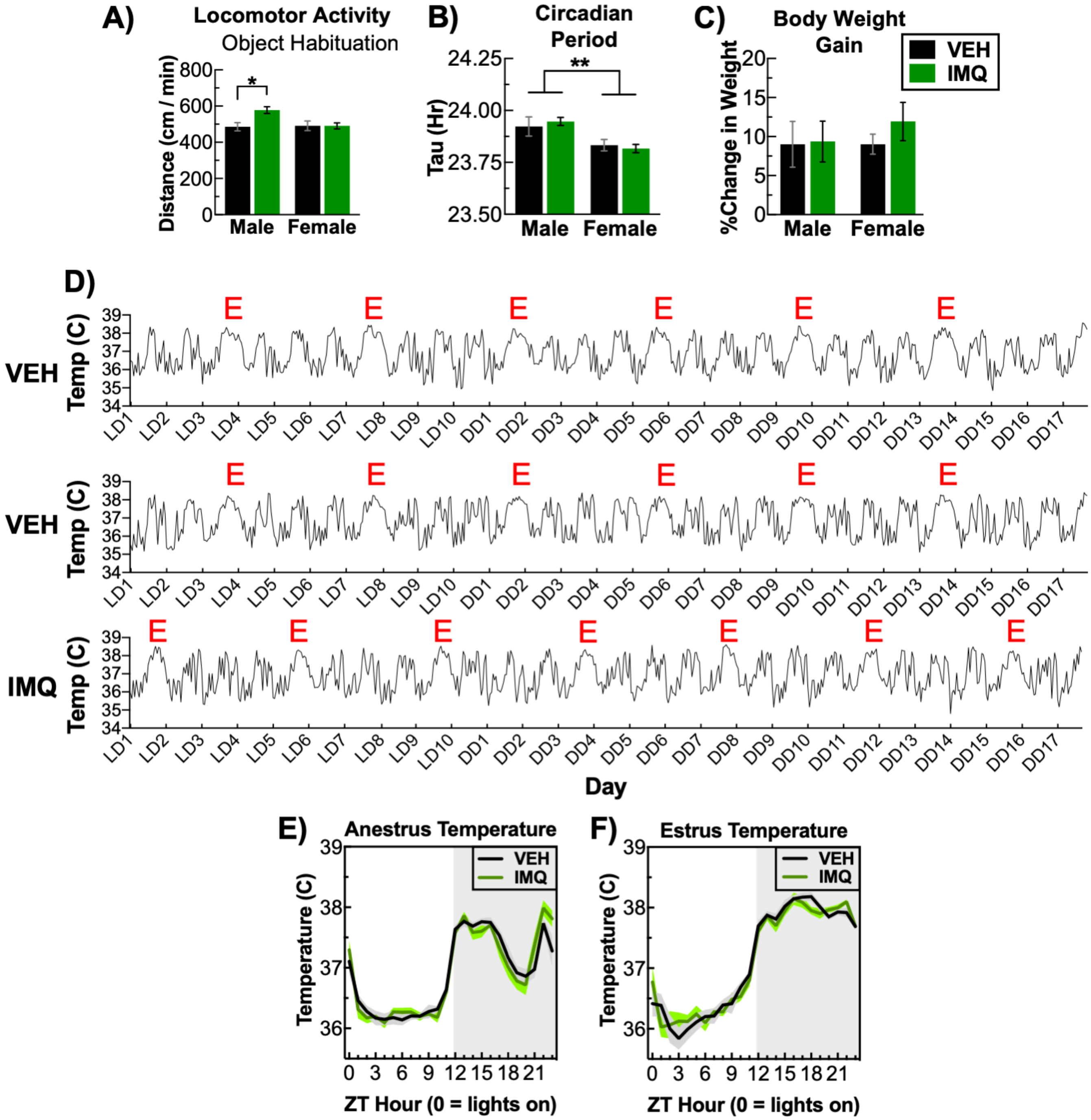

Supplemental Figure 4

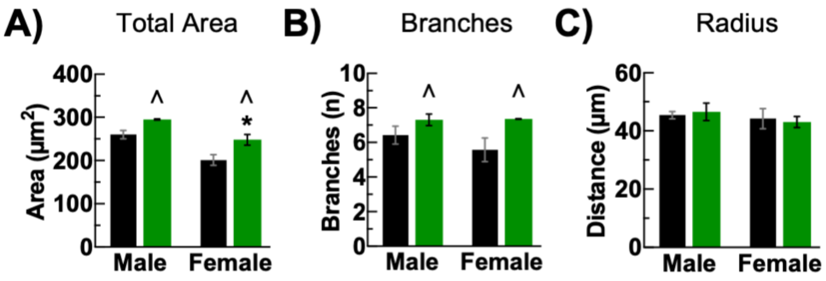

Supplemental Figure 5

A)

Differentially Expressed Transcripts

Shared Male and Female

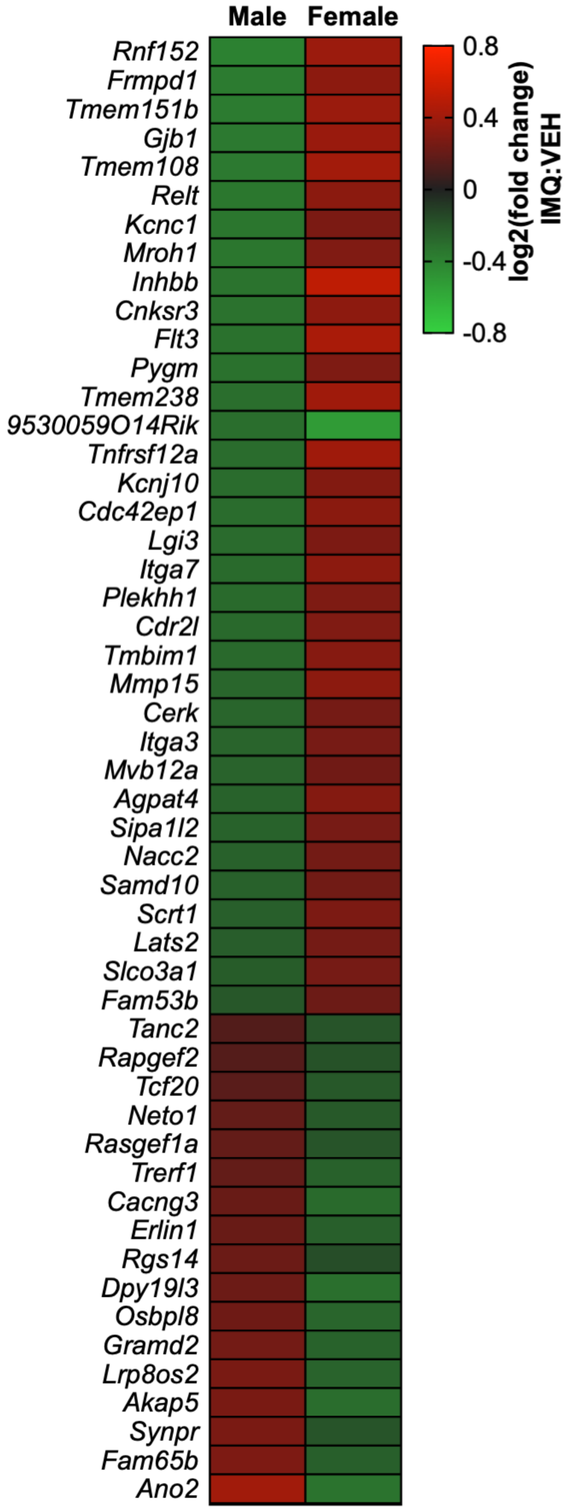

B)

Gene Ontology

Shared Male and Female

Males - Up	Males - Down
**Females - Up**	**Females - Down**
Ras protein signal transduction	cation channel complex
	ion channel complex
	transmembrane transporter complex
	transporter complex
	voltage-gated ion channel activity
	voltage-gated channel activity
	gated channel activity
	axon part
	cation channel activity
	channel activity
	passive transmembrane transporter activity
	ion channel activity
	substrate-specific channel activity
	voltage-gated cation channel activity
	potassium channel activity
	potassium channel complex
	cell leading edge
	voltage-gated potassium channel complex
	leading edge membrane
	neuron projection membrane
	potassium ion transport
	plasma membrane receptor complex
	axolemma
	potassium ion transmembrane transport
	cellular potassium ion transport
	neuromuscular junction

Males - Up	Males - Down
**Females - Down**	**Females - Up**
anchoring junction	postsynapse
adherens junction	asymmetric synapse
cell-cell junction	neuron to neuron synapse
potassium ion transport	postsynaptic density
monovalent inorganic cation transport	postsynaptic specialization
	synaptic membrane
	postsynaptic membrane
	regulation of synaptic plasticity
	axon part
	modulation of chemical synaptic transmission
	cell leading edge
	cytoplasmic region
	filopodium
	terminal bouton
	cognition
	dendrite morphogenesis
	small GTPase mediated signal transduction

Supplemental Figure 6

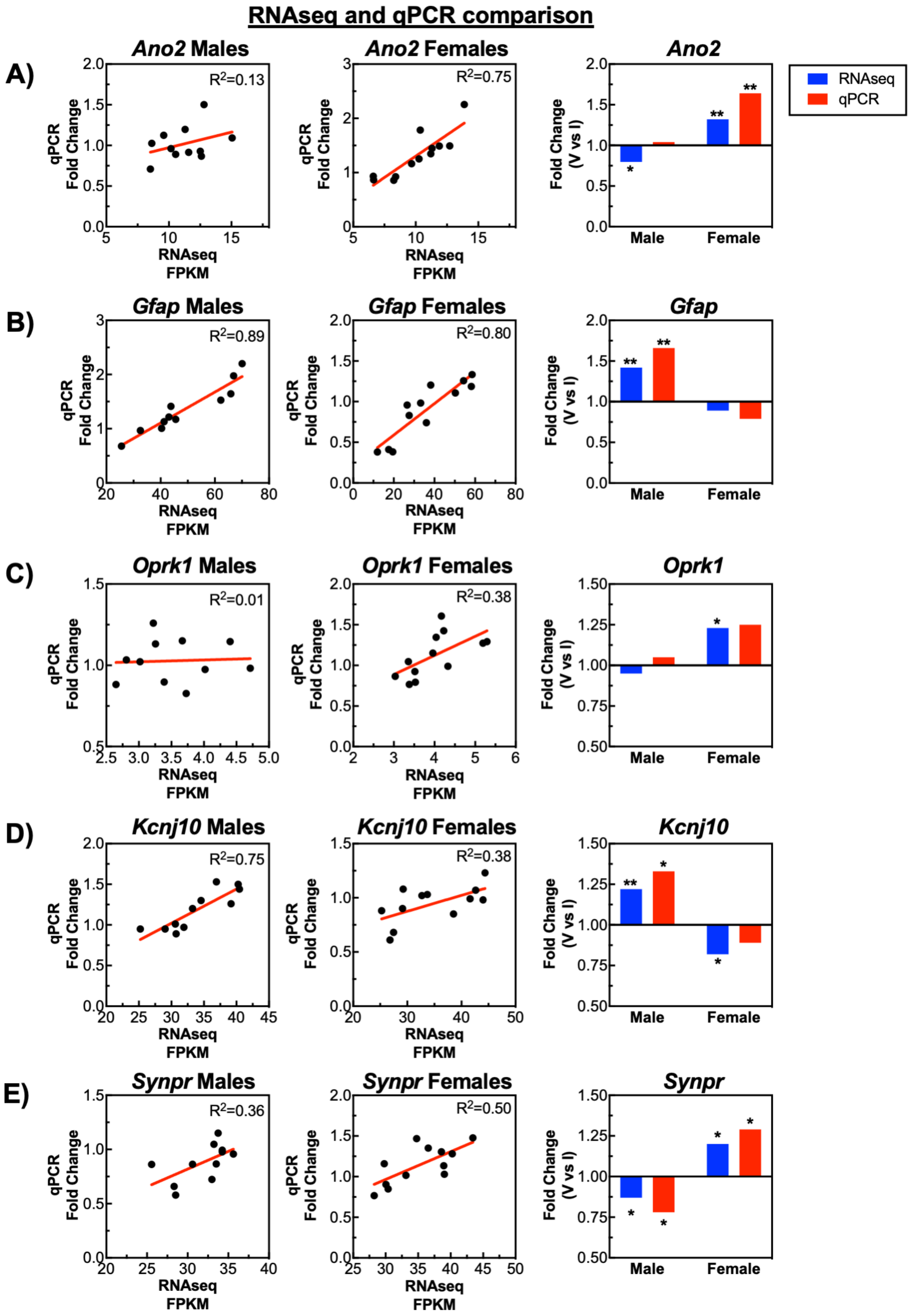

Supplemental Figure 7

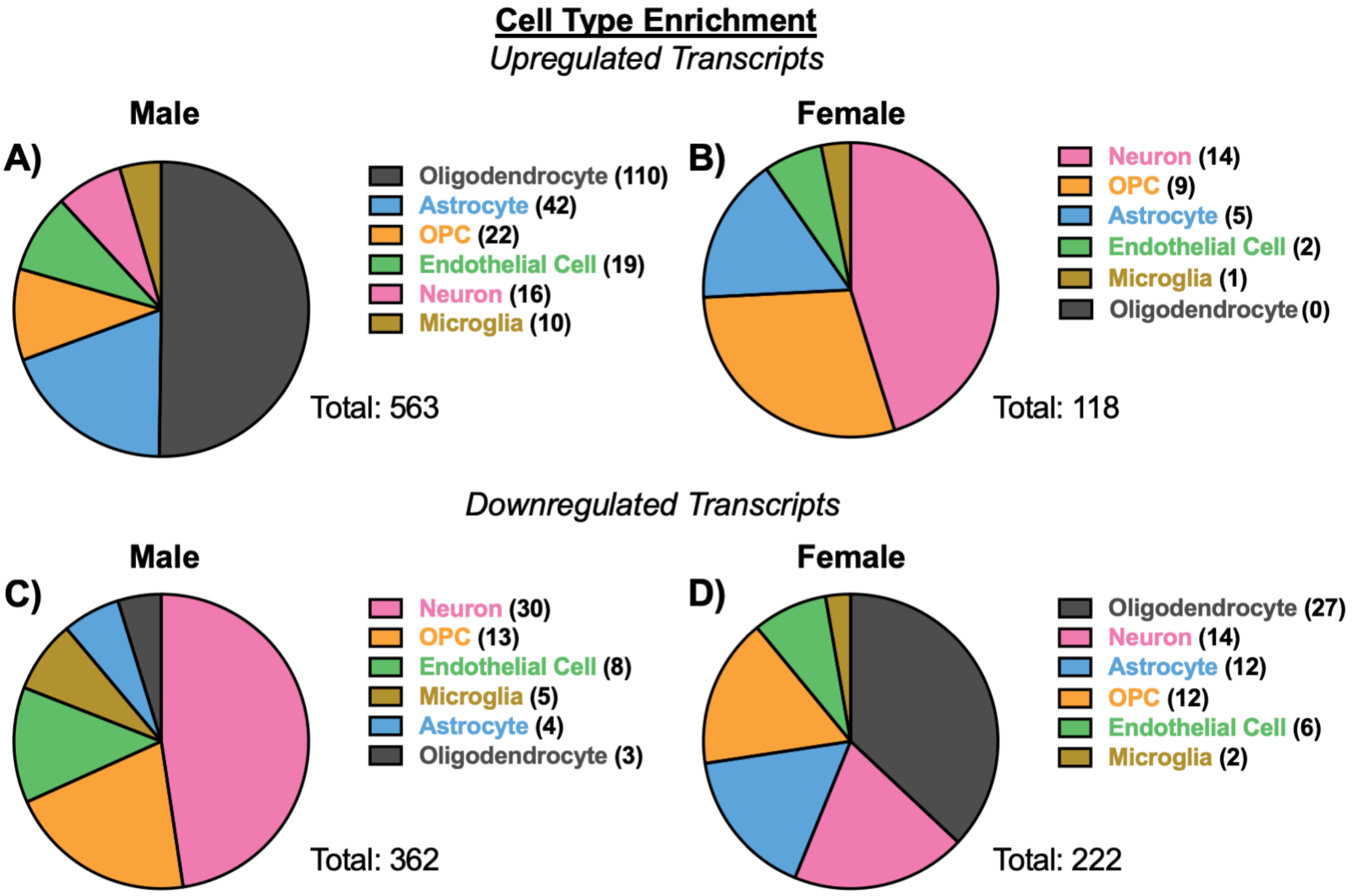

Supplemental Figure 8

Cell-type specific expression analysis

Males

A) *Upregulated*

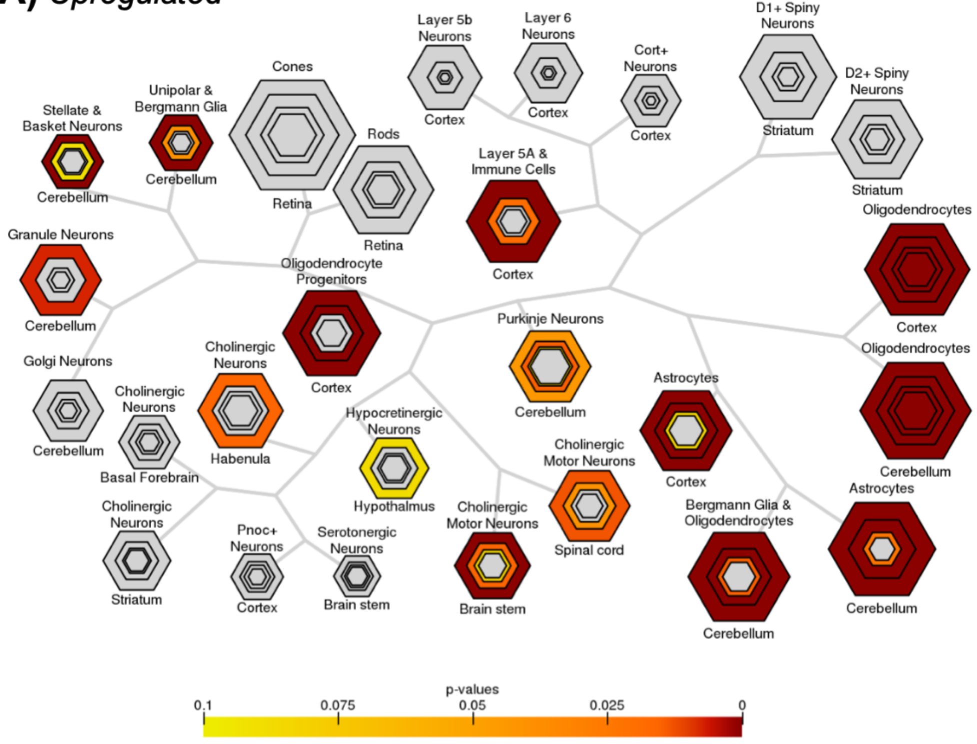

B) *Downregulated*

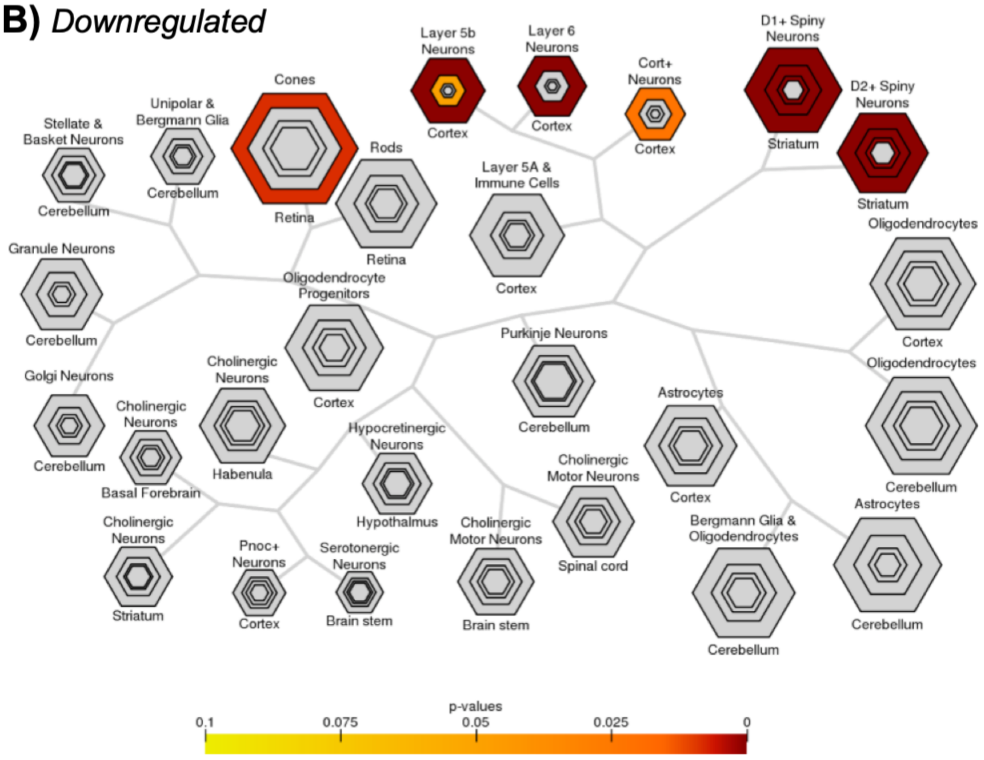

Supplemental Figure 9

Cell-type specific expression analysis

Females

A) *Upregulated*

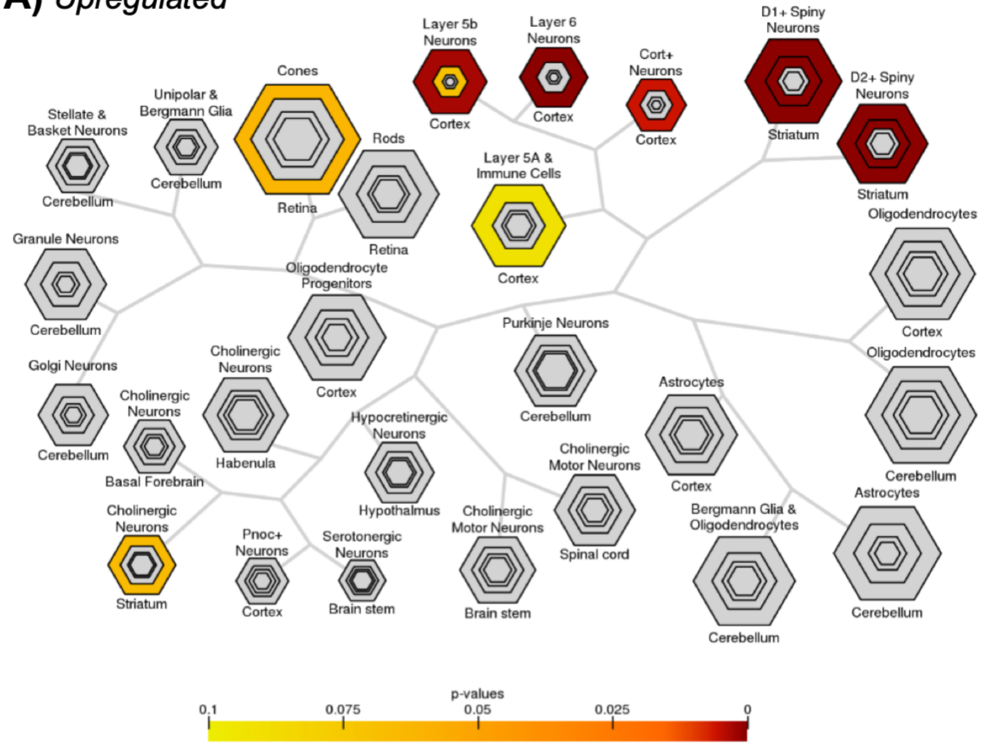

B) *Downregulated*

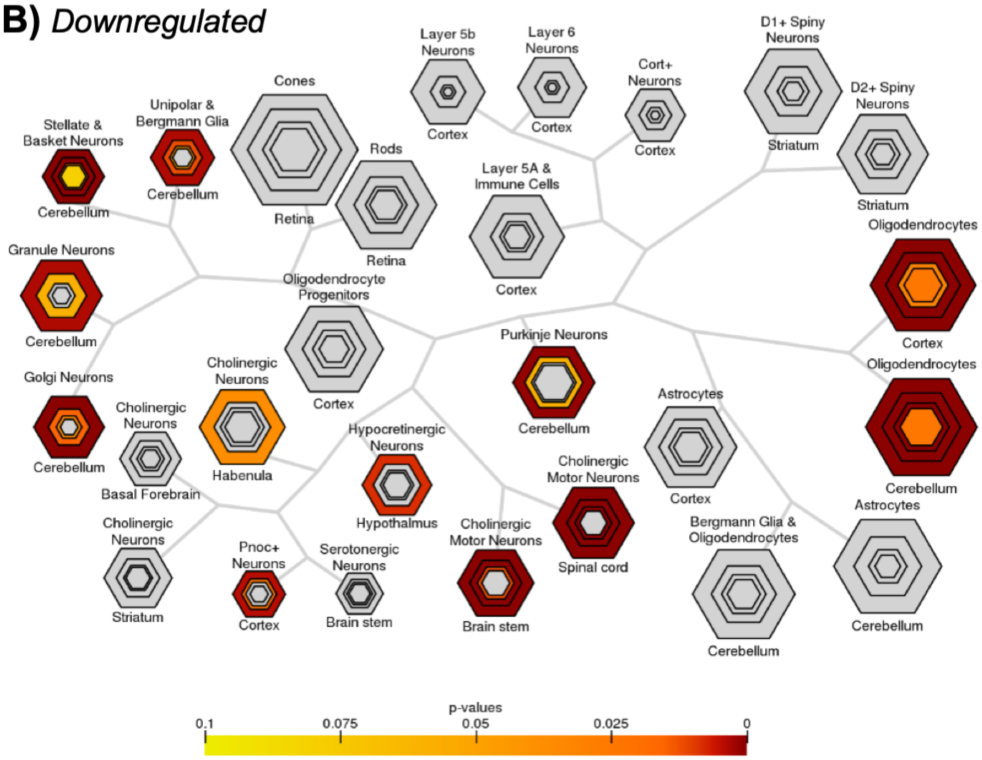

Supplement: Supplementary file 2 — Supplemental Figures [file 41380_2018_346_MOESM2_ESM.pdf]
